# Comprehensive emergency management of obstetric and newborn care program implementation at University of Gondar Comprehensive Specialized Hospital, Northwest Ethiopia, 2021: an evaluation study

**DOI:** 10.1186/s12978-023-01623-x

**Published:** 2023-05-19

**Authors:** Melak Jejaw, Ayal Debie, Lake Yazachew, Getachew Teshale

**Affiliations:** 1 Teda Health Sciences, Teda, Ethiopia; 2 Department of Health Systems and Policy, Institute of Public Health, College of Medicine and Health Sciences, University of Gondar, P.O. BOX: 196, Gondar, Ethiopia

**Keywords:** Comprehensive emergency obstetric and newborn care, University of Gondar Comprehensive Specialized Hospital, Ethiopia

## Abstract

**Background:**

Maternal healthcare service is the care given for the woman during her gestation, delivery and postpartum period. The Maternal Mortality Ratio (MMR) was remains high and a public health problem in Ethiopia. Sub-Saharan African (SSA) countries account two-thirds of the global total maternal deaths. To curb such high burden related with child births, comprehensive emergency obstetric care is designed as one of the strategies for maternal healthcare services. However, its implementation status was not well investigated. This study aims to evaluate the implementation of comprehensive emergency obstetric and new born care program in terms of Availability, compliance and acceptability dimensions at University of Gondar Comprehensive Specialized Hospital, Northwest Ethiopia.

**Methods:**

A single case study design was employed from 01 to 30 April 2021. A total of 265 mothers who gave birth at University of Gondar Comprehensive Specialized Hospital (UoGCSH) during the data collection period for acceptability, 13 key informant interviews (KIIs), 49 non-participatory observations (25 observations during C/S performance and 24 assisted spontaneous vaginal deliver) and 320 retrospective document review were conducted. Availability, compliance and acceptability dimensions were evaluated using 32 indicators. Binary logistic regression model was fitted to identify factors associated with acceptability of the services. Adjusted Odds Ratio (AOR) with 95% confidence interval (CI) and p-value < 0.05 were also used to identify associated variables with acceptability. The qualitative data were recorded using tape recorder, transcribed in Amharic and translated to English language. Thematic analysis was done to supplement the quantitative findings.

**Results:**

The overall implementation of comprehensive emergency obstetric and newborn care (CEmONC) was 81.6%. Moreover, acceptability, availability and care provider’s compliance with the guideline accounted 81, 88.9 and 74.8%, respectively. There were stocked-out of some essential drugs, such as methyldopa, nifidipine, gentamycin and vitamin K injection. CEmONC training gaps, inadequate number of autoclaves, shortage of water supply and long-distance delivery ward to laboratory unit were also the barriers for the CEmONC service. Short waiting time of clients (AOR = 2.40; 95%CI: 1.16, 4.90) and maternal educational level (AOR = 5.50, 95%CI: 1.95, 15.60) were positively associated with acceptability of CEmONC services.

**Conclusion:**

The implementation status of CEmONC program was good as per our judgment parameter. Compliance of healthcare providers with the guideline was fair and needed improvement. Essential emergency drugs, equipment and supplies were stocked-out. The University of Gondar Comprehensive Specialized Hospital was therefore had better to give great emphasis to expand maternity rooms/ units. The hospital had better to avail the resources and provide continuous capacity building for healthcare providers to enhance the program implementation.

## Plain Language Summary

Maternal healthcare service is the care given for the woman during her gestation, delivery and postpartum period. The Maternal Mortality Ratio (MMR) was remains high and a public health problem in Ethiopia. Sub-Saharan African (SSA) countries account two-thirds of the total global maternal deaths. To curb such high burden related with child births, comprehensive emergency management obstetric and newborn care is designed as one of the strategies for maternal healthcare services. The implementation status of CEmONC program service at University of Gondar comprehensive specialized hospital was good as per the preset judgment parameter. Unavailability of resources such as delivery couch, operational table, maternity and labor ward beds, glove, gauze, blood, vital sign instruments, and essential drugs including methyldopa, nifidipine, gentamycin, and vitamin K were stocked out and the challenges to provide CEmONC services. Healthcare providers’ compliance with the implementation protocol were also fairly affected. Moreover, acceptability CEmONC service was also judged as good as per the judgmental evaluation parameter. Hospital had better to fulfil the necessary equipment and drugs to enhance the implementation status of the hospitals. Capacity building of healthcare providers might also a better strategy to improve the compliance. Strengthening awareness creation for women and their husbands had a paramount importance to enhance the acceptability of the services.

## Background

Maternal health is the health of woman during her gestation, delivery and after delivery through providing prenatal care, intra-partum and post-partum care, respectively [1]. Maternal mortality is still high and a global public health problem with Maternal Mortality Ratio (MMR) of 211 per 100,000 live births. Sub-Saharan African (SSA) countries account two-thirds (40%) of the global MMR [2, 3]. Maternal mortality was also a public health problem in Ethiopia with a MMR of 412 per 100,000 live births at the end of millennium development goals (MDGs) [4]. The direct obstetric causes including hemorrhage, obstructed labor, ruptured uterus, pregnancy induced hypertension, puerperal sepsis and unsafe abortion accounted 70% of the total maternal deaths in Ethiopia [4, 5]. Majority of maternal deaths were avoidable through intervening an intensive management of complications during pregnancy, labor and delivery, and postpartum [6]. About 15% of mothers’ experienced severe obstetric complications and needs comprehensive emergency obstetric and newborn care (CEmONC) services in Ethiopia [7].

The Ethiopian government set a 5 years health sector transformation plan (HSTP) in 2015/16 to reduce the MMR to 199 per 100,000 live births and avert such high maternal deaths in 2020 [8, 9]. The World Health Organization (WHO) tried to design different policy and program to realize the global MMR lower than 70 per 100,000 live births by the end of 2030 [4, 10]. An CEmONC was one of the strategies to achieve this goal and it has a package of service necessary to manage the direct obstetric complications [11]. It comprises the seven fundamentals of basic emergency obstetric and newborn care (BEmONC) interventions, including parenteral antibiotics, uterotonic drugs, parenteral anticonvulsants, manual removal of placenta, removal of retained products of conception, assisted vaginal delivery, and resuscitation of newborn care plus blood transfusion, caesarian delivery and neonatal resuscitations [11]. CEmONC service was first launched in 1992 and had an explicitly organized guideline for the purpose of monitoring the availability and practice of the service [12]. A minimum of one CEmONC facility had planned to establish in every four BEmONC facilities and for every 500,000 population to prevent pregnancy related complications during pregnancy, labor and delivery, and postpartum period [13].

Appropriate healthcare service was very crucial to prevent obstetric complications during delivery [7]. CEmONC helps to curb maternal and neonatal mortality related with delay in receiving appropriate care at health facilities [14–16]. Although CEmONC program was implemented for a long period, only few evidences were available on its implementation status and the barriers and successes on the implementation of the program in Ethiopia. Therefore, this evaluation aims to assess the implementation status of comprehensive emergency obstetric and new born care program at University of Gondar comprehensive specialized hospital (UoGCSH) in terms of resources availability, compliance of health care providers and acceptability of the program services.

### Evaluation methods

#### Evaluation settings

The Evaluation was conducted at University of Gondar Comprehensive Specialized Hospital (UoGCSH) located in Central Gondar zone of Amhara National Regional State, Northwest Ethiopia. It is located 780 kms away from Addis Ababa (capital city of Ethiopia) and 180 kms from Bahirdar (capital city of Amhara region). The hospital established in 1954 as a public health college and training center and currently it is one of the referral and teaching hospital served for more than 7 million people in the region. The hospital had a total 66 beds in obstetric wards (9 labor beds and 57 maternity beds), two operational theaters, three maternity wards, three antenatal ward OPD, one labor ward with six delivery coach, one emergency ward and 253 Gynecology and / or obstetric staffs.

#### Evaluation objectives

The study aimed to assess the availability of required resources for the CEmONC program. Also, the level of compliance of health care providers to the national guideline during CEmONC program service delivery and the level of CEmONC client satisfaction were determined, and factors associated with CEmONC client satisfaction level were identified.

#### Evaluation approach and dimensions

**Fig. 1:**
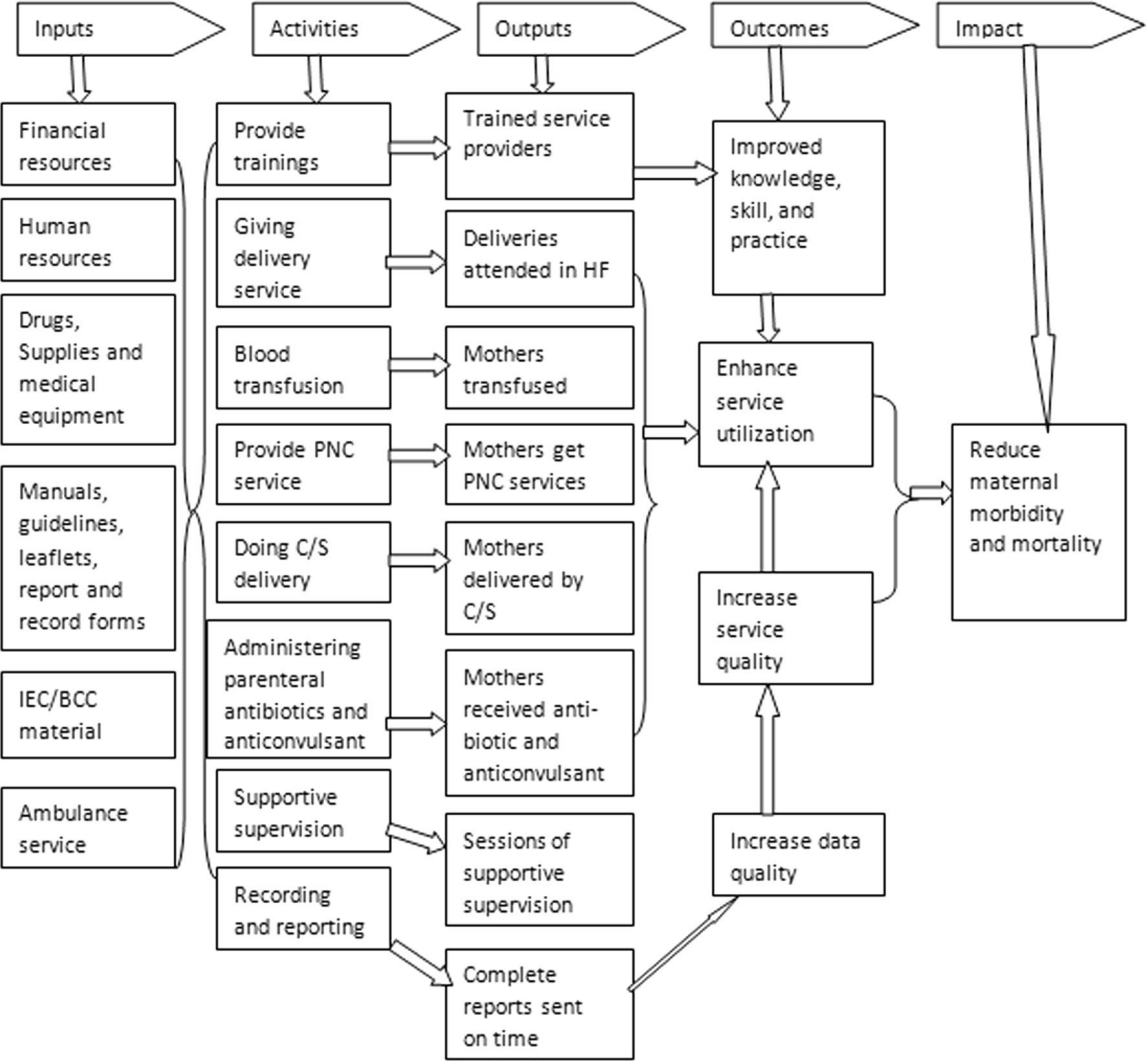
Logic model for comprehensive emergency obstetric care in UoGCSH, 2021

A formative evaluation approach with a single case study design was employed to obtain the detail and explorative reports on program implementation from 01 to 30 April 2021. The quantitative and qualitative data were collected simultaneously, analyzed separately, and mixed during interpretations of the findings. This evaluation assessed the implementation status of CEmONC program, using availability, compliance and acceptability dimensions based on the interests of stakeholders. Availability is access to health care in relation with the volume and types of services, and adequacy of resources (physical and human) used to provide CEmONC services [17]. Compliance is the degree to which CEmONC services is being implemented in UoGCSH by health care providers according to the national standards and clinical protocols [17]. Acceptability determines the level of clients (mothers who have received CEmONC service) perception about CEmONC services [17]. The focus of the evaluation was process theory which included input, activity and output (immediate result of program activities) as indicated in the program logic model (Fig. 1).

#### Sample size and sampling procedure

Availability of necessary medical equipment, supplies and medicines, and human resource and infrastructure were inspected by principal investigators by using resource inventory checklist.

Observation was conducted in delivery ward to assess the healthcare providers’ adherence to the guideline and WHO surgical safety standards. Data for compliance were collected until information saturation and based on recommendations of three or more consecutive observations to assess the compliance of one healthcare provider as per the national guideline and/or WHO standard [18]. The observation session represented the whole activity of the team and enables us to assess and judged the strength and weakness of health care providers as a whole or individual. The compliance was also assessed by using document review and the sample size was calculated by using single population proportion formula considering 50% proportion (p), 1.96 confidence interval, 5% margin of error and 10% the clients’ charts may not found. The calculated sample size was 423, but we used finite correction formula since the source population was below 10,000. We used 320 as our final sample and reviewed the maternal charts from 01 January to 01 February 2021. Purposive sampling technique was used to select the key informants based on seniority, level of education and position. The sample size for acceptability dimension was determined by using single population formula considering 79.4% patients satisfied by the service [19], 95% confidence level, 0.05 margin of error and 5% non-response rate. Our sample size for acceptability dimension was 265.

#### Data collection tools and procedures

Semi-structured questionnaire, interview guide, resource inventory, observation and data extraction checklist were prepared through reviewing of literatures [16, 18, 20]. Indicators were also developed from the national CEmONC implementation guideline, WHO surgical safety, and other related evaluations [21, 22]. Observation checklist was used to assess the healthcare provider-client interactions and providers’ adherence to CEmONC national standards, including the interpersonal interaction, ways of provider’s history taking, information transfer and other components of CEmONC as per the national standards offered to CEmONC clients. Resource inventory checklist was used to assess the existence of the required resources used for CEmONC service. This tool contains infrastructures, human resources, laboratory diagnostic tests, essential drugs, and medical equipment/supplies for CEmONC services. Interview guide was prepared for key informants, such as chief executive officers (CEOs), clinical director, midwifery head, health care providers, maternity service coordinator and Gondar town MCH coordinator and focused to assess availability and compliance. Data extraction checklist tool was prepared to review the specific procedure of diagnostic and type of signal functions of CEmONC services from clients’ chart. To find the clients’ chart, we took the medical record number from the service register. Semi-structured interviewer administered questionnaire contained the background characteristics, reproductive history, accessibility and acceptability of the service was used to assess the acceptability of the service by the clients. The questionnaire and interview guideline were translated in to the local language (Amharic) and then translated back to English language to ensure consistency.

Drug store, labor and delivery ward, laboratory room, operation theater room and the whole physical working environment via the resource inventory checklist was assessed to evaluate the availability of resource needed for CEmONC service. Besides observation, the availability of resources was also checked through interviewing the head of MCH (maternal and child health) ward and chief executive manager of the hospital. Exit interview was carried out for mothers who gave childbirths to assess the acceptability of the services. Direct observation was conducted at delivery service and operation theater room after the principal evaluator got an informed consent from the managers. Two MSc midwife students wore sterilized operational gowns and conducted a hidden observation after we got informed consent from the coordinators. A total of nine key informant interviews (KIIs) were conducted by principal evaluator after getting informed consent from interviewees. Key informants were interviewed using the interview guide and probing was done following their response to receive more information. Tape recorder and taking field notes were required during the interviewing process. The interview was taken about 20–30 min for each KIIs.

#### Data quality control

Two days training was given for data collectors about the basic techniques of data collections. Pre-test was also conducted on 13 participants at Poli health center, a neighboring affiliated health center where Caesarean delivery and blood transfusion service had provided and necessary modification was made. Observation, data extraction and resource inventory checklists were pretested and amendments were made. The questionnaire was checked for its completeness on the daily basis by the principal evaluator and supervisors during data collections. The principal evaluators transcribe the voice of the respondents in to text to analyze and check the consistence of the information with the initials. Key informant interviews (KIIs) were conducted to explore their experiences on how they deliver CEmONC services in relation with the WHO CEmONC guideline until saturation of information. Observation of healthcare providers conducted caesarian delivery in the operation theatre room to see the operation team adherence to WHO surgical safety standards by using checklists [20]. The healthcare providers did not know who observed them and it was done by two masters of Science in midwifery students who had clinical attachment in the hospital after principal evaluator had got permission from the hospital manager.

#### Data management and analysis

The quantitative data were cleaned and checked for completeness, consistency and coded by the trained supervisor and principal evaluator. Data were entered into Epi data version 4.4.1 Software and exported to SPSS version 20 for analysis. Availability, compliance and acceptability dimensions for the implementation status of CEmONC program were evaluated and judged as very good, good, fair and poor. Acceptability of CEmONC service was measured by a five point Likert scales (1 = strongly disagree, 2 = disagree, 3 = neutral, 4 = agree, and 5 = strongly agree) and classified as acceptable if the score was above or equal to the value calculated from the demarcation threshold formula $\left(\frac{\mathbf{Total \, highest}-\mathbf{Total \, lowest}}{2}\right)+\mathrm{total \, lowest}$ score [16]. Binary logistic regression model was fitted to identify the factors associated with acceptability of the services. Variables with p-value less than 0.25 during bi-variable analysis were the candidates for multivariable logistic regression and in multivariable logistic regression those variables having p-value < 0.05 were considered as the significant predictors for acceptability of the services. The tape-recorded qualitative data were transcribed and translated into text format and finally thematic analysis was done.

#### Judgment matrix

The weight of each dimension of CEmONC program was determined by the agreement of the stakeholders based on the degree of relevance. Value was given for each dimension proportionally according to their level of importance considered by the stakeholders. The score of each dimension was aggregated to decide the level of performance of CEmONC program based on the predetermine judgment criteria. Indicators’ weight is the weight given by stakeholders before the evaluation for each selected indicator, and indicator scores were calculated using the formula (Indicator score = $\frac{\mathrm{observed \, number \, X \, indicator \, weight}}{\mathrm{expected \, number}}$) [17]. The weighted values of availability, compliance and acceptability were 25%, 40% and 35%, respectively. The judgment parameter for each dimension and the overall program implementation were also categorized as poor, fair, good and very good with the corresponding judgment values of less than 60%, 60–74.9%, 75–84.9%, and more than 85%.

## Results

A total of 265 participants with a response rate of 100%, 320 clients’ chart reviews, 13 key informant interviews, and 49 client-provider interaction observations had been included for this evaluation.

### Availability

The hospital had a 24 h electric power in operation theater room with backup generator, autoclaves, functional labor and maternity ward, latrine and blood bank. The hospital had two operational tables, six delivery couches, nine labor beds and fifty-seven maternity beds. The labor and maternity beds were not proportional with number of delivery cases in the hospital. Significant numbers of mothers were laboring on the chairs and gave birth on the floor due the shortage of delivery couch and laboring beds. There were a total of 294 health professionals at Gynecology and Obstetrics (Gyn/Obs) department in University of Gondar comprehensive specialized hospital. Of these, about 8.3%, 23.3%, 65.2%, and 3.2% were Gynecology and Obstetricians, Gynecology and Obstetrician resident students, midwives, and anesthetist nurses, respectively. Only 8.7% of the care providers were received CEmONC training and there was no specific radiology, medical laboratory and environmental professionals assigned for Gyn/Obs department alone. Trained human power is the key to deliver CEmONC service to users. Absence of clear standardized selecting mechanism of health care providers for CEmONC training and lack regular CEmONC training were the barriers of implementation in the hospital. The Current clinical practice is based on evidence-based medicine approach that needs continuous and an intensive training of health professionals to harmonize with up-to-date guidelines. Key informant interviews also revealed that there is shortage of HCPs and most health care providers were not taking CEmONC trainings.

“The signal functions of CEmONC were performed with the resource that we had. It is difficult to say there were complete availability of all supplies and emergency drugs; but we use different options to deliver the service; however, it is hard to say quality service was provided to the clients” (a 30 years male third year Gyn/Obs resident).

“Not only lack of regular comprehensive emergency maternal obstetric and neonatalcare training; but some health care workers had got a chance to take the training more than once while others didn’t get even once. In addition, there is also a problem of documentation the training status health professionals. There are 165 midwifery professional staffs, but only 15 of them trained for CEmONC, this is scarcely surprising.” (29 years male midwife).

“It is obvious that there is continuous updating of guide lines thanks to advancement of science and research. It is difficult to fully believe that a mother should not die in childbirth without trained health care providers about CEmONC. I think it is better to train every health care provider to update their knowledge and skills as to fully deliver CEmONC service and to minimize the obstetric complications. Most health care providers including me did not take the pre-service and in-service CEmONC training.” (30 years male midwife).

“University of Gondar comprehensive specialized hospital has partnership with abroad country universities and few obstetricians were took short term trainings in those universities. Although there is training gaps especially for midwifery health professionals in this hospital, we have plan to use most of the working budget for obstetric care by trained the providers and to practice based on contentious health professional development approach to train the providers, to harmonize with up-to-date guide lines via refreshment trainings.” (33 years male hospital manager).

The resource inventory assessment indicates that the University of Gondar comprehensive specialized hospital had basic functional equipment and drugs needed for emergency obstetric care with full electric power supplied operational theater with back-up generator. However; there were non-functional water pip and no incinerator during our observation. The key informant interview showed that there were intermittent shortage of disposable glove and gauze, and stocked-out of some emergency drugs like methyldopa, Nifidipine and gentamycin.

“In our hospital most basic emergency drugs and supplies are stocked-in due to the existence of pharmacist drug information system. Each service unit coordinators reported to pharmacy department before two weeks of stock-out of drugs. The pharmacist also reports to Gondar branch Ethiopian Drug and Supply Agency (EDSA) before three months of stocked-out. In addition, we received some emergency drugs and supplies directly that found from central supply agency. After essential drugs reach to the hospital quality assurance committee check the quality, quantity and expire date of drugs before storage. Even though drugs are available continuously, there were stocked-out of some emergency drugs in the hospital due to missing during reporting, shortage of drugs in the central drug and supply agency.” (33 years male hospital manager).

“There were stocked-out of disposable and surgical glove in our hospital since Covid-19 pandemic rise. Even if there is an access currently, still there is on and off availability of disposable gloves overall the country due to the effect of global covid-19 pandemic. Even though maternity service is an exempted service sometimes clients may purchase gloves in private pharmacy by out of pocket due to shortage of gloves.” (33 years male maternity coordinator).

“Even though there were continuous availability and functionalities of necessary emergency drugs in maternity drug store, there were stock out of essential drugs such as methyldopa, nifidipine and gentamycin from March to April 2021.” (30 years female third year resident).

Medical instruments and equipment in obstetric ward such as sterilized delivery sets, delivery couch, labor and maternity beds, gowns, vacuum extractor and forceps, suction tube, oxygen cylinder, antiseptic, sterilized linens, partograph, different stitches and disposable container were 100% available. Vital sign measuring equipment such as blood pressure apparatus, clinical thermometer, stethoscope, and gynecological examination spot light source were not available. The KIIs reported that shortage of delivery couch, operational table, maternity and labor beds, clean towels, sterilized delivery sets and gowns were the challenges of the ward.

“University of Gondar comprehensive specialized hospital is the only public referral hospital in the town and there is over case flow in the hospital. Even though there is 24 h running operational theater and delivery services, availability of delivery couch and beds was not proportional with patient flows. As result, sometimes mothers may stay on delivery couch until they are discharge and may sit on chairs during laboring and give birth on the floor.” (31 years male third year Gyn/Obs resident).

“Since the hospital is referral and teaching hospital, many clients came from all directions to this hospital for delivery service. As a result, the hospital is overcrowded and we faced shortage of delivery couch, beds and operational table. To alleviate this burden, the hospital and the regional health bureau, in particular, should collaborate to reduce the number of direct and referral patient flow to this hospital by enhancing the capacity of the health center and primary hospital with human power and resource.” (33 years male hospital manager).

“Even though the hospital is run by federal government and we did not have any regular supportive supervision, we are provided CEmONC service beyond our working capacity. We are still working to scale up the affiliation health center site to be well equipped to provide blood transfusion and cesarean section delivery service in order to reduce direct and referral case to the hospital. The possible reasons to increase patient flow to this hospital, which cause work overload, were lack of trained health care provider at health center and primary hospitals, lack of resource in the facility, most mothers want to delivery at the referral hospital than health center and lack of delivery room at health center.” (40 years female maternal and child officer).

Based on resource inventory, the hospital had laboratory room for CEmONC service and fully functional central supply blood bank which has a capacity to store too much blood unit. The laboratory capacity had available and functional equipment and supplies such as blood type and cross matching reagent, centrifuges, refrigerator, blood unit and register materials, but there were shortage of reagents like syphilis and hepatitis (Table 1). The key informant interview showed that there were interruptions of CBC reagent, blood collection tube, blood and mal-functionality of CBC and chemistry machine in the hospital for one month.

“There was shortage CBC reagent, blood collection tube and mal-functionality of CBC and chemistry machine for one month from March to April. Physicians were unable to done complete blood count cell and organ function tests. Due to this, the physicians were forced to prescribe antibiotic medications based on physical examination finding without knowing blood cell count status” (35 years male midwife).

“The hospital has a common laboratory service which was located far from the obstetric ward. Due to absence of independent laboratory service room at the maternity ward, health care providers took blood samples to the laboratory room and return to get the results. This was very tedious for professionals and sometimes it may delay the procedures, for example during blood transfusion and cesarean section for blood grouping and compatibility. Still, we are repeatedly requesting to have laboratory service room at the obstetric ward like maternity drug store.” (30 years male third year Gyn/Obs resident).

**Table 1:** Overall judgment of availability of resources for comprehensive emergency obstetric care in University of Gondar comprehensive specialized hospital, 2021

Dimensions with indicator	E*	O*	W*	S*	A* (%)	JP*
Proportion of gynecologist available in the hospital	6	12	2.5	5	100	Very good
Proportion of health workers received training on CEmONC program	100	22	3.3	0.73	22.1	Poor
Proportion of Ambo-bag available in delivery room	5	5	2.5	2.5	100	Very good
Percentage of essential drugs available for comprehensive obstetric care	21	20	3	2.9	96.7	Very good
Proportion of delivery coach in delivery room	10	6	3.3	2	60.6	Fair
Proportion of blood unit available in the hospital for mothers	11,000	9700	2.8	2.5	89.3	Very good
Proportion of available operation table in the hospital	8	2	3	0.8	26.7	Poor
Proportion of available suction machine in operation room	2	3	2.3	3.5	100	Very good
Proportion of available sterilized cesarean delivery sets	12	12	2.5	2.5	100	Very good
Overall availability CEmONC program resource			25.2	22.4	88.9	V. good

*E* expected, *W* weight, *O* observed, *S* Score ((observed × weight)/Expected), *A* achievement in percentage ((S/W) * 100), *JP* judgment parameter

### Compliance of HCPs (client-providers interaction observation result)

The total of 49 direct observations (25 in operation room and 24 in the labor and delivery) were conducted (to assess their level how they provide CEmONC services as per the national guideline and WHO surgical safety standards. More than three-fourths (76%) of health care providers introduced themselves and call the clients by their names. Two-thirds (68%) attended the labor and delivery through partograph and measured their vital sign and Forty percent (40%) of them reviewed the women’s chart about their previous history of pregnancy and birth outcome before any procedure. Eighty percent (80%) of the clients were informed about any pregnancy related complication and their managements. Only one-thirds (34%) of the operation theater team introduced their name and role to the clients during cesarean section. Over ninety five percent (95.8%) of cesarean section delivery had been done based on indication. Moreover 92% of women received prophylaxis antibiotic an hour before the cesarean section. All clients’ blood group and Rh factor were done, but X-match was performed only for 40% of them. Only half (50%) of the healthcare providers were strictly followed WHO surgical safety checklist standards during performing operation (Table 2). The key informant interviews (KIIs) revealed that caesarean section delivery without clear indications becomes common in this hospital.

“Performing caesarean section delivery without clear indication due to fear of complication becomes common in this hospital. It is my fear that no mother will give birth vaginally in the future if caesarean section delivery without clear indication continued in this manner.” (29 years male midwife).

“I had working in this hospital for many years. I had notice that conducting cesarean section delivery increase time to time. Occasionally, I do believe there are some problems of regarding to the indication of cesarean section in this hospital. Even if the reasons were not clearly well known, in my experience the reason may be due to fear of complication by residents. If complications happen the duty resident might think they will lag or suspend from resident ship promotion so they may create false indication and underwent cesarean section even the case might have a chance to be managed by other options. Other reasons may be due to low senior consultation involvement and cesarean section becomes fashion now a day.” (35 years male midwife).

**Table 2:** Direct observation result of delivery service by health care providers in University of Gondar comprehensive specialized hospital, 2021

	Performed by care providers Frequency (%)
Tasks for pregnant mother performed in labor ward (n = 25)	
Check for the availability of washing facilities (water, soap, towel)	3 (12)
Greets and calls client by her name and introduce herself/himself/	19 (76)
Reviews patients record before the procedure and check previous obstetric history	10 (40)
Provider used partograph to follow labor	17 (68)
Take pulse rate, blood pressure, temperature, respiratory rate	17 (68)
Provider document information on partograph and registers	18 (72)
Do neonatal resuscitation performed based on algorithm	11 (100)
Informs mothers about her and fetus’s health condition	24 (96)
Informs mothers about any complication and management	20 (80)
Records all findings, assessments, diagnosis, and care with clients	23 (92)
Laboratory investigation requested for mothers	
Coagulopathy test (platelet counts)	9 (36)
Urine analysis	16 (64)
Serum blood sugar test	6 (24)
HGB/HC	19 (76)
X-match	16 (64)
Blood group and RH factor	25 (100)
HIV test	12 (48)
Tasks performed by operation teams in operation room theatre (n = 24)	
Mother whose surgical safety check list filled during caesarean section	12 (50)
Has the patient confirmed his/her identity, site, procedure, and consent?	23 (95.8)
Is the site marked?	10 (42)
Is the anesthesia machine and medication checked complete?	24 (100)
Is the pulse oximetery on the patient and functioning?	21 (88)
Does the patient have a known allergy?	4 (17)
Difficult airway or aspiration risk	8 (34)
If yes, equipment/assistance available	8 (100)
Confirm the patient’s name, procedure, and where the incision will be made	14 (59)
Has antibiotic prophylaxis been given with in the last 60 min?	22 (92)
Anticipated blood loss	17 (71)
Has sterility been confirmed?	23 (96)
Are there equipment issues or any concerns?	9 (38)
Nurse verbally confirms the procedure, completion of instrument, sponge and needle counts, specimen labeling addressed any equipment problems	18 (75)
Are there any key concerns for recovery and management of this patient?	10 (42)
Confirm all team members have introduced themselves by name and role	8 (34)

### Performance of CEmONC signal functions (clients’ chart review results)

The past 2-month clients’ chart were reviewed and the results showed that all signal functions were presented. Relatively the most frequently performed signal functions were administration of parenteral uterotonic (40.4%), administration of parenteral antibiotic (17.14%) and cesarean section delivery (12.1%). However, neonatal resuscitation (8.3%), parenteral anticonvulsant (6%), manual removal of placenta (5%), blood transfusion (4%) and removal of retained product (2%) were the least implemented signal functions. The key informant interview results showed that the nine signal functions were performed within the past 2 months even though there were on and off existence of supplies and medicines. HIV test of mothers came for CEmONC service was neglected.

“Nationally one of the Maternal and child health goals was achieving HIV free generation. To achieve this goal every mother should be screening for HIV. Based on the report we received from university of Gondar comprehensive specialized hospital, all clients were not screened for HIV. We tried to communicate with the hospital manager and HMIS focal person because all clients were not screened for HIV. I believe that either the care provider did not screen for HIV or there might be poor recoding and reporting system.” (40 years female maternal and child officer).

The common obstetric complication encountered in the hospital in the past 2 months was ante-partum hemorrhage (13%) and obstructed labor (11.2%), puerperal sepsis (6.5%), postpartum hemorrhage (6%), Eclampsia (6%), premature rupture of membrane (3.4% and rupture of uterus (1%). All obstetric complication was managed in the hospital and the mother’s life was saved (Table 3).

**Table 3:** Summary analysis and judgment matrix of compliance for evaluation of comprehensive emergency obstetric care program in University of Gondar comprehensive specialized hospital, 2021

Dimensions with indicator	E	O	W	S	A (%)	JP
Proportion of mothers who gave birth by caesarean section based on indication	25	24	3.6	3.5	97.22	Very good
Proportion of early birth neonate resuscitated based neonatal resuscitation algorithm	11	11	3.2	3.2	100	Very good
Proportion of mothers who informed about any complication and management	25	20	3.2	2.6	81.25	Good
Proportion of health professional who greet and call by name mothers	25	19	3.6	2.7	75	Good
Proportion of mothers whose vital sign measured	25	17	4	2.7	67.5	Fair
Proportion of pregnant mothers whose partograph filled	25	17	5.2	3.5	67.3	Fair
Proportion of mothers whose surgical safety checklist filled during C/S	24	12	3.6	1.8	50	Poor
Proportion of pregnant mothers whose previous pregnancy history and birth outcome checked before procedure	25	10	4	1.6	40	Poor
Proportion of pregnant mothers whose X-match is done	25	16	2.8	1.8	64.3	Fair
Proportion of pregnant mothers whose blood group and Rh factor done	25	25	3.2	3.2	100	Very good
Proportion of pregnant mothers who received antibiotic prophylaxis within the last 60 min before c/s done	24	22	3.6	3.3	91.7	Very good
Overall compliance of CEmONC service provider		24	40	29.9	74.8	Fair

*E* expected, *W* weight, *O* observed, *S* Score ((observed × weight)/Expected), *A* Achievement in percentage ((S/W) * 100), *JP* judgment parameter

### Acceptability of CEmONC services

#### Socio-demographic characteristics

Half (52.8%) of the respondents were under the age of 20 years and the mean age was 28.5 years (± 5.54 SD). Most respondents (89.1%) were married and 50.2% of them were house wives. More than half (57.4%) of the respondents were attended secondary education and above, and 66% of the respondents had average household monthly income more than 1000.00 ETB. Two-thirds (67.9%) of the participants were urban dwellers and 79.2% of them were Orthodox Christian followers.

#### Obstetric characteristics of participants

More than half (58.5%) of the women were multi-gravid and 60.8% were primipara. The major mode of deliveries for the current pregnancy in this hospital was spontaneous vaginal delivery (57.3%) and ninety percent of (90.9%) of the birth outcomes were live births. Nearly one-fourth (26.8%) of clients gave births at hospital before current pregnancy. More than ninety percent (95.8%) of the clients were also willing to receive blood if she had indication (Table 4).

**Table 4:** Obstetric characteristics of the participants in University of Gondar comprehensive specialized hospital, 2021 (N = 265)

Obstetric history	Category	Frequency (n = 265)	%
Gravidity	Prime-gravid	110	41.5
	Multi-gravid	155	58.5
Parity	Prime para	161	60.8
	Multi para	104	39.2
Mode of delivery	Spontaneous vaginal delivery	153	57.7
	Cesarean section delivery	98	37
	Instrumental delivery	14	5.3
Birth outcome	Alive	241	90.9
	Still birth	5	1.9
	Other	19	7.2
Frequency of hospital visit	One times	42	15.8
	Two times	52	19.6
	Three times	80	30.2
	Four times	43	16.2
	More than four	48	18.1
Reasons for hospital visit	Pregnancy test	13	4.9
	ANC follow up	25	9.4
	HIV testing	14	5.3
	To give birth	66	24.9
Facility for ANC follow-up the recent pregnancy	At this hospital	91	34.3
	At another hospital	37	14
	At health center	96	36.2
	At health post	34	12.8
	No ANC visit	7	2.6
Number of ANC visits for recent pregnancy	One times	26	9.8
	Two times	50	18.9
	Three times	82	30.9
	Four times	82	30.9
	More than four	25	9.4
Previous delivery before current pregnancy	At hospital	71	26.8
	At health center	63	23.8
	At home	27	10.2
Willing to receive blood	Yes	254	95.8
	No	11	4.2

#### Health facility related characteristics

More than one-third (39.6%) of clients were travelled for half an hour to receive the CEmONC services. Nearly two-thirds (61.9%) travelled by public transport and 9.8% went to the hospital on foot. About 60% of women were spent more than an hour with the health care provider during service provision.

#### Acceptability of CEmONC

The overall acceptability of CEmONC services was 81%. Almost all (98.9%) mothers were accepted the healthcare providers explanation about the treatment they received. More than 95% of participants also accepted the discussion and understand about pregnancy related danger sign and pain management during delivery care, ANC, and consultation time. Over 85% of women accepted the waiting time to receive the CEomNC services at the hospital. Nearly 65% of women reported the visual privacy during examination were acceptable and half (52.8%) of them also accepted the auditory privacy (Table 5). The key informants revealed that clients were complained in related to waiting space, physical abuse, humiliation, absence of family support during labor and visual privacy. *“Most mothers were complained to waiting space or area cleanness and absence of family support, visual privacy during vaginal examinations disrespect of maternal care and humiliation by care provider.” *(a 30 years female third year resident student)*.*

**Table 5:** Overall performance of maternal acceptability for comprehensive emergency obstetric care program in University of Gondar comprehensive specialized hospital, 2021

Dimensions with indicator	E	O	W	S	A (%)	JP
Proportion of pregnant mothers satisfied to explanation provided or treatment given	265	262	2.8	2.76	98.9	Very good
Proportion of pregnant mothers satisfied to waiting time	265	230	3.5	3.04	86.9	Very good
Proportion of mothers satisfied to health care provider open mindedness or greeting them while treating them	265	252	3.2	3.04	95.1	Very good
Proportion pregnant mothers satisfied to visual privacy during examination	265	171	2.8	1.8	64.3	Fair
Proportion pregnant mothers who satisfied to the cleanliness of the delivery room	265	249	3.2	3	85.7	Good
Proportion pregnant mothers satisfied to auditory privacy during discussion	265	140	2.8	1.5	53.6	Poor
Proportion of mothers satisfied to pain management	265	260	3.2	3.14	98.1	Very good
Proportion of mothers satisfied for consultation time	265	260	3.2	3.14	98.1	Very good
Proportion of mothers satisfied to the confidentiality of health care provider	265	258	2.5	2.4	96	Very good
Proportion of mothers satisfied for the waiting area safety and cleanses	265	27	3.5	0.4	11.4	Poor
Proportion of mothers satisfied with discussion about pregnancy related danger sign	265	261	2.1	2.06	98.5	Very good
Proportion of mothers satisfied to ANC follow up service	265	260	2.5	2.4	96	Very good
Overall acceptability of CEmOC service			35.3	28.68	81	Good

*E* expected, *W* weight, *O* observed, *S* Score ((observed × weight)/Expected), *A* Achievement in percentage ((S/W) * 100), *JP* judgment parameter

### Factors associated with acceptability of CEmONC

In multi variable logistic regression waiting time for service and educational status of women were variables associated with acceptability of CEmONC services. Accordingly, women who had waiting time of less than an hour to receive the CEmONC services were 2.40 times (AOR = 2.40; 95%CI: 1.16, 4.90) more likely accepted the services as compared with women who waited for one or more hoursto receive the services. Non-educated mothers were 5.50 times (AOR = 5.50; 95%CI: 1.95, 15.60) more likely accepted the CEmONC services as compared with women who have secondary and above educational level (Table 6).

**Table 6:** Factors associated with acceptability of CEmONC service in University of Gondar comprehensive specialized hospital, 2021

Variables	Category	Acceptability		COR	95% CI		AOR	95% CI		p-value
		Yes	No							
Maternal education	Unable to read and write	40	22	0.28	0.12	0.56	5.5*	1.95	15.6	0.002
	Read and write	10	4	0.38	0.11	1.32	2.8	0.6	13.2	0.21
	Primary	33	4	1.25	0.4	3.90	0.8	0.22	2.6	0.64
	Secondary and above	132	20	1			1			
Waiting time	< 60 min	76	30	0.37	0.19	0.69	2.4*	1.16	4.9	0.02
	≥ 60 min	139	20	1			1			
No. of ANC visits	One	24	1	5.65	0.72	44.25	0.15	0.17	1.34	0.09
	Two	39	8	1.15	0.47	2.83	0.57	0.19	1.68	0.31
	Three	63	18	0.82	0.4	1.68	1.12	0.5	2.5	0.79
	Four and above	85	20	1			1			
Parity	Prime Para	137	24	1.90	1.02	3.54	0.74	0.29	1.9	0.53
	Multi Para	78	26	1			1			
Time taken to the hospital	< 30 min	63	13	1.67	0.77	3.6	0.61	0.24	1.56	0.3
	30–60 min	90	15	2.10	1.01	4.35	0.66	0.27	1.6	0.35
	> 60 min	63	22	1			1			
Gravidity	Primigravida	94	16	1.65			1.05	0.39	2.8	0.92
	Multigravida	121	34	1			1			

*Significant in multivariable regression at p-value < 0.05 and 95% confidence interval

#### Overall judgment matrix

The overall implementation status of CEmONC was 81.6% and judged as good. Availability, compliance and acceptability of CEmONC services were 88.9, 74.8 and 81% and judged as very good, fair and good as per the preset judgment parameter, respectively (Table 7).

**Table 7:** Overall judgment matrix and analysis of dimension for CEmONC program in University of Gondar comprehensive specialized hospital, 2021

Dimensions	Weight (%)	Achievement (%)	Judgment parameter
Availability	25	88.9	Very good
Compliance	40	74.8	Fair
Acceptability	35	81	Good
Overall dimensions	100	81.6	Good

## Discussion

The overall implementation status of CEmONC was 81.6% and judged as good. It was calculated as the average of availability, compliance and acceptability dimension findings. Moreover, availability, compliance and acceptability of CEmONC services were 88.9, 74.8 and 81% and judged as very good, fair and good as per the preset judgment parameter, respectively. The finding indicated that the overall evaluation and acceptability of the service needs an improvement and mainly compliance of healthcare providers required an urgent improvement. Availability of resources was relatively very good. The availability of resource for CEmONC service at University of Gondar comprehensive specialized hospital was 88.9%. This result was higher than the overall availability of resource in Shenan Gibe hospital (73.8%) [20]. The discrepancy might be due to working set up variation, stock balance controlling mechanism and differences in hospital management bodies. Only 23.8% of gynecologist and obstetrician and 9.1% of midwives received CEmONC training. This was comparable with 2016 Ethiopian emergency obstetric and newborn care (EmONC) report which was only 7% midwives had comprehensive EmONC [23] and CEmONC training in Shenan Gibe Hospital, Jimma, Ethiopia [20].

Stocked-out of emergency drugs like methyldopa, nifidipine and gentamycin, syphilis and hepatitis reagents, disposable gloves and gauze were also reported in the hospital. Evaluation study conducted in Shenan Gibe hospital revealed that absence of disposable glove and gauze were observed [20].Although most (96.7%) essential drugs for CEmONC service was available some emergency drugs were stocked out for the past 2 months. The possible reasons for such stock-out might be due to shortage at central store, inadequate transport and administrative difficulties. Shortage of sterilized delivery set and gowns which was in line with the 2016 Ethiopian emergency obstetric and neonatal assessment reports [23]. According to our evaluation finding, incinerator, gynecological examination light, laryngoscope, mechanical ventilator, umbilical catheter, endotracheal intubation and vital sign equipment like blood pressure apparatus, thermometer and stethoscope were absent in the delivery ward. Our finding was lower than the Ethiopian emergency obstetric and neonatal care assessment reports [24].

Shortage of water supply, linen sets, delivery couch, operation couch (table), maternity and labor beds and blood unit were also observed. Evaluation study was conducted in Shenan Gibe and Ghana, Nigeria and Sera Leone showed that there were shortage of lack of blood and absence of operational theater room [20, 23, 25]. This dedicated that unavailability of basic medical equipment and supplies were the factors hindering to deliver the signal functions of CEmONC services.

Adherence of health care providers to the national guideline CEmONC program implementation was 74.8%. Blood group and Rh factor investigations were done for all clients. This finding was consistent with the recommendation of the national guideline that every pregnant mothers had got blood group and Rh factor tests [26]. However, it was slightly higher than similar evaluation in Shenan Gibe hospital (95%) [20]. Most (95.8%) of cesarean section was done based on clear indications and informed consent of the clients. This was higher than the evaluation done in Shenan Gibe hospital (60%) [20]. This variation might be expert differences. UOGCSH had better experts since it is a teaching hospital and it might have plenty of specialized and sub-specialized physicians. As a result, the physicians might exercise the WHO’s declaration that increasing the rate of caesarean sections did not have an extra advantages on reduction of maternal and newborn mortality [27].

Most (92%) of the CEmONC service during observation was administrations of prophylactic antibiotic before an hour cesarean section. This finding was supported by a systematic review conducted on antibiotic prophylaxis versus no prophylaxis for preventing infection after cesarean section [28] and the WHO surgical safety standards recommendation [22] in the review report and WHO recommendation indicated that administration of a prophylactic antibiotics before cesarean section substantially reduce the risk of infections. Two-thirds of women in labor (68%) attended by using partograph and measuring vital signs. This finding was consistent with an evaluation study done in Shenan Gibe hospital (70%) [20]. This low partograph uptake might be due to high work load, lack of motivation, and shortage of staffs [29]. Consistency of the operational team with surgical safety checklist was only 50% in the current evaluation. This finding was similar an evaluation conducted in Shenan Gibe hospital, Ethiopia (40%) [20], but WHO surgical safety checklist standards recommends that any operational teams sought to be complete the surgical safety checklist standards before underwent any surgical procedure. The reasons for non-adherence to standards were absence of cooperation among operation teams and lack of training on surgical safety standards.

Verification of patient identity before provision of the service and the presence of operation theater team members were only 40 and 34%, respectively. This finding was incongruent with a systemic review report on compliance and perceptions of care providers to surgical safety checklist standards adherence to the confirmation of patient identity and procedure (90%) [30] and WHO surgical safety standards[22]. The discrepancy may be due to lack of staff motivation, low expected cooperation, and weak leadership and staff awareness. All basic signal functions were available and functional at University of Gondar comprehensive specialized hospital. Our finding fulfils the national success of the health facility’s CEmONC standards [31].The most implemented signal functions in this hospital were administration of parenteral urotonic (40.4%), administration of parenteral antibiotics (17.1%), and cesarean section delivery (12.1%). However, the least practiced basic signal functions were administration of parenteral anticonvulsant (6%), assisted vaginal delivery (4.9%), blood transfusion (4%) and removal of retained product (2%). These findings were in line with a study conducted in Shenan Gibe hospital, Ethiopia [20] and the 2016 Ethiopian EmONC survey reports [23].

Cesarean section delivery (12.1%) was within the range of UN recommendation (5–15%) and slightly higher than an evaluation study done Hadya zone (7.1%) [32]. Antepartum hemorrhage (13%) and obstructed labor (11.2%) while puerperal sepsis (6.5%), postpartum hemorrhage (6%), premature rupture of membrane (3.4%) and rupture of uterus (1%) commonly recorded obstetric complications encountered in the hospital. Training of healthcare providers on CEmONC program was only 8.7% in the present study. The possible reasons for low training were diversity of training bundles, lack of resource and absence of evidence-based medical practices [33].

The overall acceptability of CEmONC service provision was 81% as per the preset judgment parameter. This result was similar with an evaluation study conducted Shenan Gibe hospital (82.9%), Assela hospital (80.7%), public health facilities in Jimma zone (79.4%) and Mekelle hospital (79.7%) [20, 34–36]. However, the finding was lower than findings at Arba Minch town (90.2%) and Gamo Gofa zone [37, 38]. Nearly all mothers (98.9%) had high acceptance about healthcare providers’ explanation about the treatments. This finding was higher than evaluation conducted at Shenan Gibe hospital (67.9%) [20]. The justification for this discrepancy might be due to an improvement in quality service provisions, variations in the anticipation of mothers and work setups. Visual privacy during examination, auditory privacy during discussion, and safety and cleanliness of waiting area were 64.5, 52.8 and 10.2%, respectively. Visual and auditory privacies were comparable with a study done in public health facilities in Shenan Gibe hospital [20], Referral hospitals of Amhara national regional state [16, 39] and national and WHO standards [40]. The possible reasons for such low acceptance related with visual and auditory privacies might be due to shortage of curtains, negligence of care provider, removal of clothing without consent, provide a clothes that does not cover the whole bodies of the women, and absence of private room for delivery.

Non-educated mothers were more likely accepted the CEmONC services compared with the educated women. This finding was also supported by a study in Kaffa, Ethiopia [41] and Australia [42]. The possible reasons might be due to educated mothers were more ambitious and high expectation with maternal care services. Mothers who had spent less than an hour with healthcare providers were more likely accepted the services compared with their counterparts. This finding was supported by a study conducted in Ethiopia [43]. The possible justifications might be due to longer stay in ward could affect mother’s needs family care and cultural factor and they feel safety when they are at home.

## Limitations

This evaluation focused only on availability, compliance and acceptability of CEmONC implementation. The qualitative data collections (observation) may also prone to hawthorne effects and to reduce this effect the first three observations were excluded from analysis. Weighting of each dimension and indicator were given by stake holders and this might prone to bias. To minimize this bias the stakeholders reviewed the standards and detail discussion was held. However, this evaluation could not address the other dimensions. The regression was done for only acceptability dimension due to feasibility issue. We recommend for other evaluators to address these limitations in future studies.

## Conclusion

The overall implementation status of CEmONC program service at University of Gondar comprehensive specialized hospital in terms of availability, compliance and client satisfaction dimensions was judged as good as per the preset judgment parameter. Although the required resource is available adequately, this program implementation needs an improvement. Paucity of delivery couch, operational table, maternity and labor ward beds, sterilized delivery set and gown, gynecological spot light, glove, gauze, blood, vital sign instruments, wall clock and incinerator were frequently observed in the hospital. Methyldopa, nifidipine, gentamycin, vitamin K injections, reagents for syphilis and hepatitis were also stocked out. Poor compliance on utilization of partograph, maintaining of WHO surgical safety standard, measuring vital sign, blood X-match, verification of patient identity and operational team members before procedure. Overall acceptance of mother with the process of CEmONC service was judged as good. Strengthening awareness creation for women and her husband had a paramount importance to enhance the acceptability of the services. Capacity building of healthcare providers might also a better strategy to improve the compliance. Hospital had better to fulfill the necessary equipment’s and drugs to enhance the implementation status of the hospitals. Further researches shall be done including other dimensions of process evaluation such as affordability and accommodation to improve the implementation status. Outcome and impact evaluation shall be conducted to evaluate its effect on morbidity and mortality of women.

## Data Availability

Data will be available upon reasonable request from the corresponding author.

## Abbreviations

ANC: Antenatal care
ARH: Amhara regional health
BCC: Behavior Change Communication
BEmONC: Basic Emergency Obstetric and Newborn Care, BP: Blood Pressure
CBC: Complete Blood Count
CEmONC: Comprehensive Emergency Obstetric Newborn Care
CS: Cesarean section
IESO: Integrated Emergency Surgical Officer
MCH: Maternal and Child Health
MDGs: Millennium Development Goals
MMR: Maternal mortality ratio
SDGs: Sustainable development goals
SVD: Spontaneous vaginal delivery
UoGCSH: University of Gondar Comprehensive Specialized Hospital
